# Ubiquitination and dynactin regulate TMEPAI lysosomal trafficking

**DOI:** 10.1038/srep42668

**Published:** 2017-02-20

**Authors:** Shenheng Luo, Lei Jing, Tian Zhao, Yuyin Li, Zhenxing Liu, Aipo Diao

**Affiliations:** 1 School of Biotechnology, Tianjin University of Science and Technology, Key Lab of Industrial Fermentation Microbiology of the Ministry of Education, Tianjin, 300457, China

## Abstract

The transmembrane prostate androgen-induced protein (TMEPAI) has been reported to be elevated in various tumor cells, is localized to the lysosome and promotes lysosome stability. The molecular mechanism of TMEPAI trafficking however to the lysosome is unknown. Here we report that clathrin and CI-M6PR mediate TMEPAI transport from the Golgi directly into the endo-lysosomal pathway. TMEPAI is ubiquitinated at its C-terminal region and ubiquitination modification of TMEPAI is a signal for its lysosomal trafficking. Moreover, TMEPAI binds the ubiquitin binding proteins Hrs and STAM which is required for its lysosomal transport. In addition, TMEPAI interacts with the dynactin pointed-end complex subunits dynactin 5 and dynactin 6. The aa 132–155 domain is essential for specific TMEPAI binding and deletion of this binding site leads to mis-trafficking of TMEPAI to the plasma membrane. These results reveal the pathway and mechanism regulating transport of TMEPAI to the lysosome, which helps to further understand the role of TMEPAI in tumorigenesis.

Protein trafficking in the secretory and endocytic pathways is a multistep process involving the transport of proteins from a particular intracellular or extracellular compartment to another. This is regulated by an array of pathways including membrane trafficking, protein translocation, and endocytosis or exocytosis^1^. Membrane trafficking has become an increasingly studied area of cellular machinery. The membrane bound organelles have different functions designed to facilitate protein transport and for providing distinct compartments specifically for its target proteins. In addition, the functional organization of the cell is maintained by the selectivity of the vesicular transport which plays a central role in the transport of molecules between different membrane-enclosed compartments. Lysosomes are ubiquitous organelles which function as the primary degradative compartments of cells. The integrity of the lysosome structure and its function is maintained by lysosomal membrane proteins (LMPs) and hydrolases. Lysosome biogenesis requires the involvement of both secretory and endocytosis pathways. Degradative cargo and newly synthesized lysosomal proteins target to the lysosome from an endo-lysosome system with or without passing through the plasma membrane, indicating two distinct trafficking pathways. Firstly the trans-Golgi network (TGN) feeds directly into the endo-lysosome system, and the best-characterized direct intracellular pathway is the clathrin-dependent transport of lysosomal hydrolases mediated by mannose-6-phosphate receptors (M6PRs)^2^,^3^. The second route is following the constitutive secretory pathway to the plasma membrane which subsequently reaches the lysosomes by endocytosis. Increasing evidence suggests that there are multiple TGN exits for LMPs, LMPs and that these can travel to the lysosomes through both direct and indirect pathways^4^,^5^.

Ubiquitination was originally described as a protein degradation signal to the 26 S proteasome^6^,^7^. More attention has been attracted however to the discovery that ubiquitination is also found to modulate numerous biological processes in yeast and mammalian cells, including vesicular trafficking, signaling transduction, endocytosis, cell-cycle modification, DNA damage repair and gene transcription^8^,^9^. The process of ubiquitinylation involves the sequential transfer of the evolutionarily conserved 76 amino acid protein ubiquitin, between ubiquitin-activating enzyme (E1), ubiquitin-conjugating enzyme (E2) and ubiquitin-protein ligase (E3), to the specific lysine residues of the target proteins. Proteins can either be mono, multi, or poly-ubiquitinated according to the degree of ubiquitin linkage to the lysine residues of the substrate or of ubiquitin itself^10^. The different forms of ubiquitin modification on a protein dictate its distinct functions. Poly-ubiquitination provides the main targeting signals for degradation to the proteasome, whereas the mono-ubiquitination and multi-ubiquitination, act as a sorting signal that regulate the intracellular protein trafficking from TGN to endosomes or lysosomes and endocytosis of plasma membrane proteins^11^,^12^. The function of The Nedd4 family of E3 ubiquitin ligases in regulating endocytosis and of the sorting of transmembrane proteins has been demonstrated^13^,^14^. In mammalian cells, ubiquitination of GGA3 by Nedd4 regulates the sorting of LAPTM5 from the Golgi to endosomes/lysosomes^15^.

The endosomal sorting complex required for transport (ESCRT) system is critical for the degradation of ubiquitinated proteins and comprises a major pathway for multivesicular body (MVB) formation. The ubiquitinated proteins can be recognized by intracellular proteins that contain one or more ubiquitin-binding domains, such as Hrs and STAM which are two components of the ESCRT subunit ESCRT-0, and provide an additional targeting module that promote their binding to cargo-enriched endosomes^16^. Thus, ESCRT-0 is the detection module for initiating the ESCRT pathway at endosomes^17^,^18^, and ubiquitination is a vital modification for sorting ubiquitinated cargoes into ESCRT-mediated MVB vesicles and their subsequent transfer to lysosomes^19^.

In addition to sorting signal of ubiquitination, microtubules provide the tracks for protein transporting, along which cargo is carried to its destination. Microtubule-based transport is of critical importance for the localization and motility of endomembranes. Motor proteins consisting of dynein, kinesin and myosin co-ordinate to physically move cargo along the microtubule network. Dynactin is a multisubunit protein complex that is required for dynein activity through the direct binding of dynein with p150^Glued^^20^ and allows the motor to traverse the microtubule lattice over long intracellular distances. It has been suggested that dynactin contributes to microtubule anchoring^21^ since impairment of dynactin structure or its function commonly leads to the redistribution of endomembrane compartments from the cell center to the periphery^22^,^23^. Loss of p25 (dynactin 5) significantly weakened the physical interaction between dynein and early endosomes^24^, leading subsequent redistribution of early endosome^25^.

Transmembrane prostate androgen-induced protein (TMEPAI), also known as PMEPA1 (prostate transmembrane protein androgen induced 1) or STAG1 (solid tumor-associated 1 protein), has been reported to be overexpressed in various tumor cells^26^,^27^,^28^. TMEPAI is a type Ib transmembrane protein containing 287 amino acids containing a transmembrane domain at the N-terminus and two PY motifs (PPxY) which interact with E3 ubiquitin ligase Nedd4^29^. Previous studies have shown that TMEPAI localizes to the lysosome and downregulates TGF-β signaling by competing with SARA (Smad anchor for receptor activation) for R-Smad binding to sequester R-Smad phosphorylation^30^ and promoting lysosomal degradation of TGF-β receptor (TβR)^31^. TMEPAI promotes lysosome stability and autophagy^32^. Moreover, it has been reported that the E3 ubiquitin ligase Nedd4-binding defective mutant of TMEPAI was translocated to the plasma membrane^31^. The molecular mechanism of TMEPAI trafficking to the lysosome and of E3-binding defective mutant TMEPAI (2YA) translocation to the plasma membrane is unknown. Here, we further investigated and demonstrated that clathrin and CI-M6PR mediated TMEPAI transport from the Golgi directly into the endo-lysosomal pathway, and ubiquitination modification of TMEPAI is a signal for its lysosomal trafficking. In addition, the dynactin complex is involved in the transport of TMEPAI to the lysosome.

## Results

### Clathrin and CI-M6PR modulate TMEPAI transport from the Golgi to the lysosome

**Figure 1:**
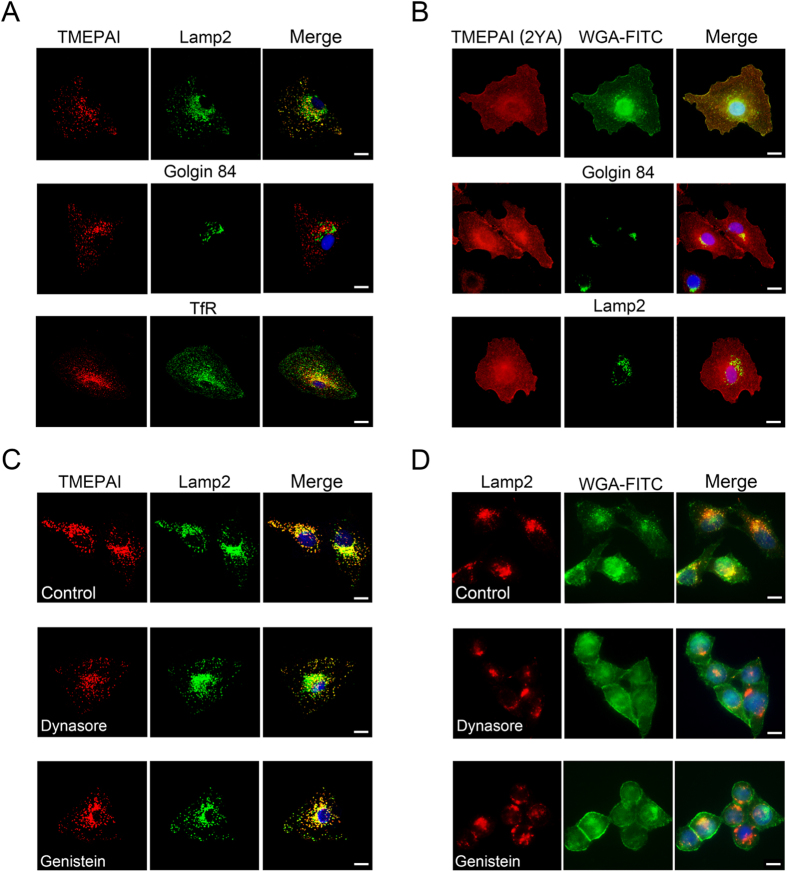
TMEPAI transports to lysosome without passing through plasma membrane. (**A**) Immunofluorescence microscopy of A549 cells stably expressing TMEPAI-Flag double labelled with antibodies to Flag (red) and either the lysosome maker Lamp2, Golgi marker Golgin-84 or recycling endosome marker transferrin receptor (TfR) (green). Scale bar, 10 μm. (**B**) A549 cells stably expressing TMEPAI-2YA-Flag were double labelled with antibodies to Flag (red) and either plasma membrane marker lectin wheat germ agglutinin labelled with FITC (WGA-FITC), Golgin-84 and Lamp2 (green). Scale bar, 10 μm. (**C**) A549 cells were treated with endocytosis inhibitor dynasore (80 μM, for 2 h) or genestein (100 μM for 6 h) before being fixed and double labelled with antibodies to TMEPAI (red) and Lamp2 (green). Scale bar, 10 μm. (**D**) A549 cells were preincubated with 80 μM dynasore or 100 μM genestein at 37 °C for 30 min, WGA-FITC was added and incubated for 2 min at room temperature. The cells were washed with medium containing dynasore or genestein respectively, and the cells were incubated for 15 min at 37 °C. The cells were fixed and double labelled with antibodies to Lamp2 (red) and WGA-FITC (green). Scale bar, 10 μm.

It has been reported that TMEPAI was mainly localized to the lysosome, while the Nedd4-binding defective mutant of TMEPAI was translocated to the plasma membrane^31^. We further verified that endogenous TMEPAI was mainly localized to the lysosome, partially overlapped with TfR and not colocalized with the Golgi (Fig. 1A), whilst the TMEPAI (2YA) was localized to the plasma membrane (Fig. 1B). Lysosomal proteins can reach the lysosome through either direct or indirect pathways which are distinguishable in whether the transport process passes through the plasma membrane or not. To determine if the intracellular transport of TMEPAI is via a plasma membrane route, the endocytosis inhibitors dynasore and genistein were used to define the trafficking pathway of TMEPAI to the lysosome. Dynasore acts as an inhibitor of endocytic pathways and depends on dynamin 2 by rapidly blocking coated vesicle formation^33^. Genistein is a tyrosine-kinase inhibitor that causes local disruption of the actin network at the site of endocytosis and inhibits the recruitment of dynamin 2^34^. Our results showed that TMEPAI remained in the lysosome with no apparent alternation after treatment with endocytosis inhibitor (Fig. 1C), and the function of dynasore and genestein in inhibiting endocytosis was verified (Fig. 1D). Furthermore, depletion of dynamin 2 by RNA interference and expression of dynamin 2 GDP mutant (K44A) also did not affect the lysosomal localization of TMEPAI (data not shown). These results indicate that TMEPAI transports to the lysosome without passing through the plasma membrane.

**Figure 2:**
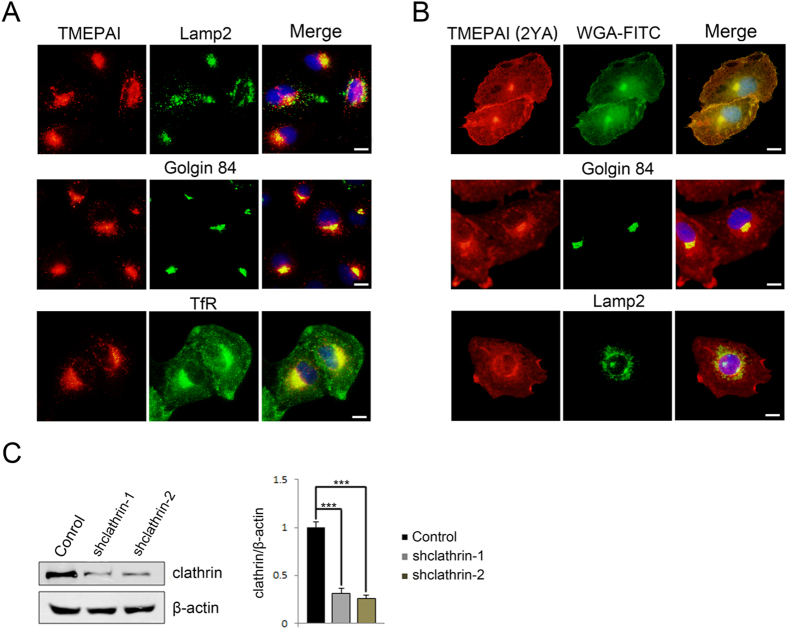
Clathrin regulates TMEPAI intracellular localization. (**A**) A549 cells infected with clathrin-shRNA lentivirus 4 days before double labelled with antibodies to TMEPAI (red) and TfR, Golgin 84 and Lamp2. Scale bar, 10 μm. (**B**) A549 cells stably expressing TMEPAI-2YA-Flag were infected by clathrin-shRNA lentivirus 4 days and labelled with antibodies TMEPAI (red) and WGA-FITC, Golgin 84 and Lamp2 (green). Scale bar, 10 μm. (**C**) Clathrin-shRNA lentivirus efficiency was accessed by Western blot with antibody against clathrin and quantitation (N = 3, ***P < 0.001).

Dynasore and genistein both act on dynamin 2, an essential protein engaged in clathrin-mediated endocytosis^35^. Clathrin serves to mediate vesicle-coating on TGN exits^36^. To determine the potential involvement of clathrin in both TMEPAI and its mutant form in sorting from TGN, clathrin-shRNA targeting the clathrin heavy chain was performed in order to deplete clathrin and block clathrin coated vesicles (CCVs) formation. Depletion of clathrin showed an enrichment of TMEPAI and its mutant TMEPAI (2YA) was observed in the Golgi labelling with the Golgi marker Golgin 84) (Fig. 2A,B), together with the redistribution to the Golgi and plasma membrane of transferrin receptor (TfR), a receptor coated by clathrin during its exit from TGN and internalization on plasma membrane, and depletion of clathrin also resulted in the redistribution of Lamp2 to the perinuclear region. Western blot analysis was used to confirm the efficient depletion of clathrin (Fig. 2C). These results indicate that TMEPAI lysosomal transport is clathrin-dependent.

**Figure 3:**
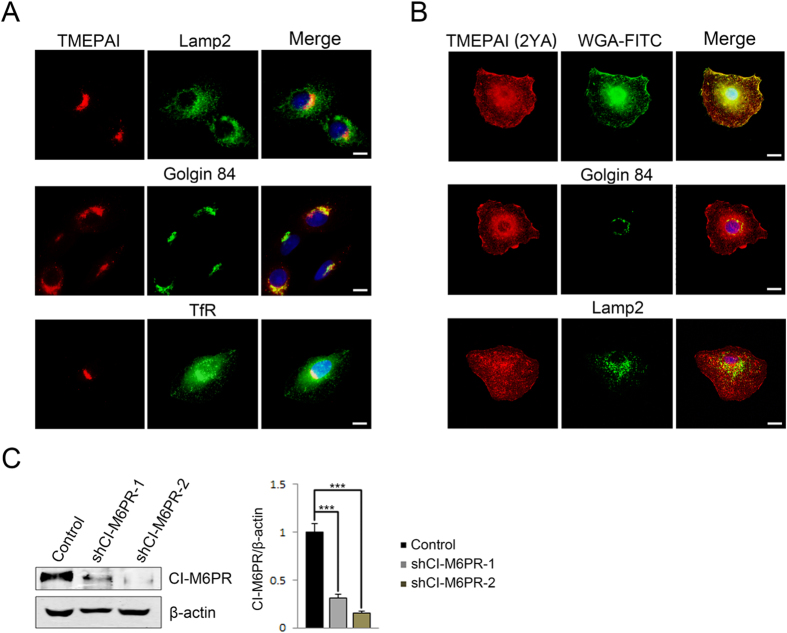
CI-M6PR involves in TMEPAI intracellular trafficking to the lysosome. (**A**) A549 cells infected with CI-M6PR-shRNA lentivirus 4 days before being double labelled with antibodies to TMEPAI (red) and Golgin 84, Lamp2 and TfR. Scale bar, 10 μm. (**B**) A549 cells stably expressing TMEPAI-2YA-Flag were infected by CI-M6PR-shRNA lentivirus 4 days and labelled with antibodies to TMEPAI (red) and WGA-FITC, Golgin 84 and Lamp2 (green). Scale bar, 10 μm. (**C**) CI-M6PR-shRNA lentivirus efficiency was accessed by Western blot with antibody against CI-M6PR and quantitation (N = 3, ***P < 0.001).

Mannose-6-phosphate receptors (M6PRs), as well as clathrin are involved in lysosomal protein trafficking. There are two types of M6PRs, cation-independent mannose-6-phosphate receptors (CI-M6PR) and cation-dependent mannose-6-phosphate receptors (CD-M6PR), both of which deliver newly synthesized acid hydrolases to the endo-lysosomal pathway and then return them to the TGN. To investigate whether M6PRs regulate TMEPAI transport, M6PR levels were reduced by M6PR-shRNA lentivirus infection. Depletion of CI-M6PR resulted in TMEPAI retention to the Golgi (Fig. 3A), whereas no effect was observed on TMEPAI (2YA) localization on the plasma membrane (Fig. 3B). The localization of control proteins TfR and Lamp2 were not affected by the depletion of CI-M6PR. The CI-M6PR RNAi efficiency was confirmed by Western blot analysis (Fig. 3C). In addition, the localization of both TMEPAI and its mutant remained unchanged on CD-M6PR depletion (data not show). Together, these results suggest that CI-M6PR modulates TMEPAI intracellular transport to the lysosome.

### Ubiquitination regulates TMEPAI lysosomal transport

**Figure 4:**
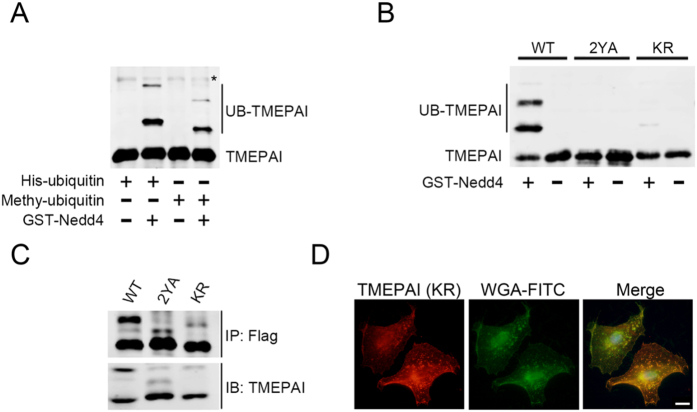
Ubiquitination modulates TMEPAI intracellular localization. (**A**) The TMEPAI recombinant protein (His-HA-TMEPAI (aa63-287)) was used for an *in vitro* ubiquitination reaction containing E1, E2, GST-Nedd4 (E3) and His-tagged ubiquitin (His-ubiquitin) or methylated ubiquitin (methyl-ubiquitin). Protein ubiquitination was analyzed by Western blot using an antibody to HA. (*Indicates non-specific band). (**B**) *In vitro* ubiquitination was performed with E1, E2, GST-Nedd4 (E3) using TMEPAI recombinant proteins: His-HA-TMEPAI-WT (aa63-287), His-HA-TMEPAI-2YA and His-HA-TMEPAI-KR. TMEPAI ubiquitination was detected by Western blot with anti HA antibody. (**C**) A549 cell lines stable expressing Flag-tagged TMEPAI-WT, TMEPAI-2YA and TMEPAI-KR were extracted and immunoprecipitated with antibody against Flag-tag, and the immunoprecipitates were analyzed by Western blot with antibody to Flag and TMEPAI. (**D**) Immunofluorescence microscopy of A549 cell lines stably expressing Flag-tagged TMEPAI-KR doubled labelled with antibody to Flag (red) and WGA-FITC (green). Scale bar, 10 μm.

Since TMEPAI sorting to the lysosome requires interaction with Nedd4^31^, the lysosomal sorting defect of the TMEPAI mutant lacking the Nedd4 binding sites is due to either blocking the TMEPAI-Nedd4 interaction or to ubiquitination modification of TMEPAI. We next tested whether TMEPAI ubiquitination is involved in its sorting to the lysosome. To determine if the ubiquitination of TMEPAI is mono- or poly-ubiquitination, His-tagged ubiquitin protein and methylated ubiquitin protein defective in mediating poly-ubiquitination were used in an *in vitro* ubiquitination assay. Both the wide-type ubiquitin and ubiquitin mutant resulted in a similar pattern of Nedd4-mediated ubiquitin ligation, which suggested that Nedd4 catalyzes mono-ubiquitination of TMEPAI (Fig. 4A). Further, we analyzed the potential ubiquitination sites on the TMEPAI protein sequence using the online ubiquitination sites prediction tool of UbiPred (http://iclab.life.nctu.edu.tw/ubipred/index.php), and found that potential ubiquitination sites presented at the C-terminal tail of TMEPAI (277-KEKDKQKGHPL-287). We then generated TMEPAI mutants TMEPAI-KR with four Lysine (K) residues mutated to Arginine (R) and produced the TMEPAI cytoplasmic recombinant protein His-HA-TMEPAI-WT, the mutants His-HA-TMEPAI-2YA and His-HA-TMEPAI-KR for additional *in vitro* ubiquitination experiments. Ubiquitination of TMEPAI-2YA and TMEPAI-KR were inhibited compared to TMEPAI-WT (Fig. 4B). Moreover, *in vivo* ubiquitination performed by immunoprecipitation of A549 cells stably expressing Flag-tagged TMEPAI-WT, TMEPAI-2YA and TMEPAI-KR showed that ubiquitination of both TMEPAI-2YA and TMEPAI-KR was disrupted (Fig. 4C). To examine whether the ubiquitination site is required for TMEPAI sorting to the lysosome, A549 stably expressing TMEPAI-KR-Flag was double labeled with anti-Flag antibody and WGA-FITC, indicating that TMEPAI-KR-Flag was present on the plasma membrane (Fig. 4D). These results indicated that ubiquitination of TMEPAI at its C-terminal is responsible for transport to the lysosome.

**Figure 5:**
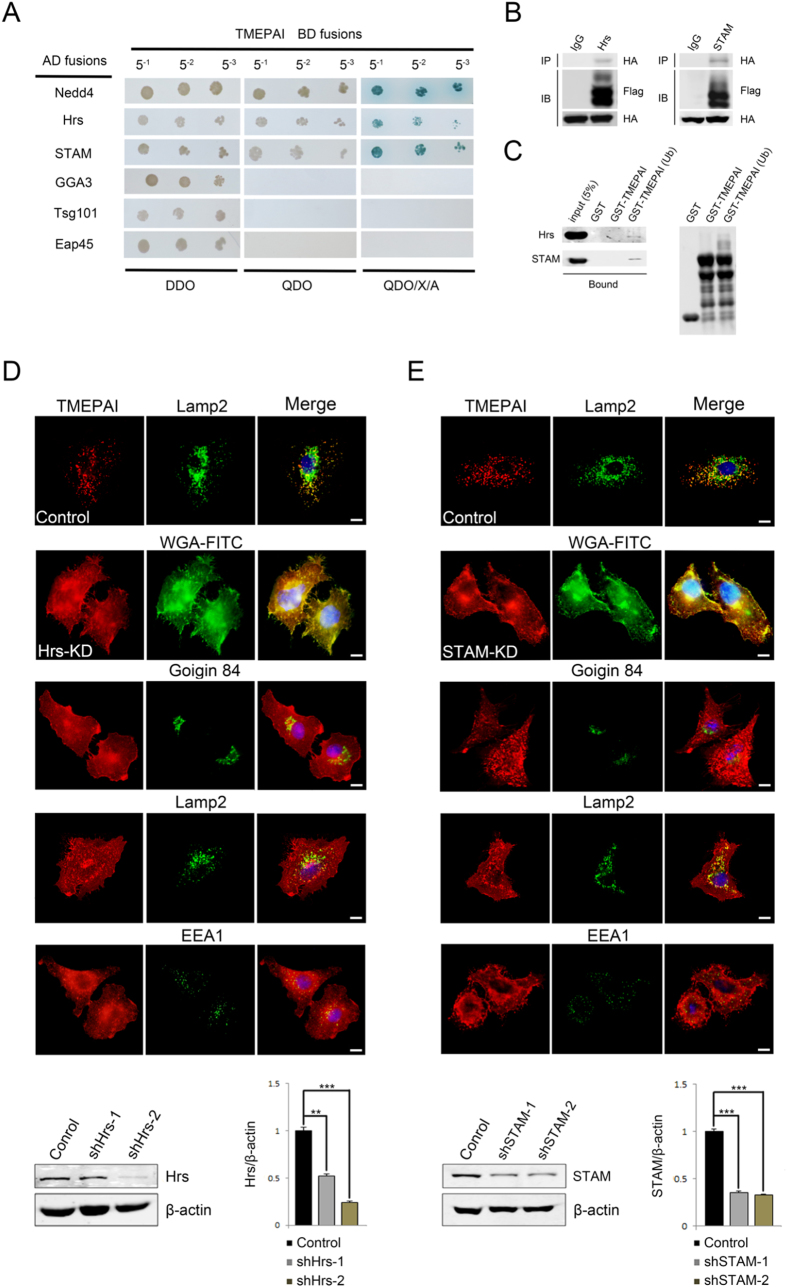
Ubiquitin binding proteins Hrs and STAM regulate TMEPAI lysosomal trafficking. (**A**) Human Nedd4, Hrs, STAM, GGA3, Tsg101 and Eap45 were fused to the GAL4 activation domain (AD fusions) and tested for interaction with the TMEPAI cytoplasmic domain (aa63-287) fused to the GAL4 DNA-binding domain (BD fusion) in the yeast two-hybrid system. Yeast diploids co-expressing GAL4 AD fusions and GAL4 BD fusions were grown in liquid selective media, diploids were titrated (5^−1^, 5^−2^, 5^−3^; total 3 μl) and patched on the DDO, QDO and QDO/X/A plates. Interaction between the indicated proteins results in growth on the QDO and QDO/X/A media. The yeast co-expressing TMEPAI and Nedd4 was used as the positive control. (**B**) HeLa cell lines co-transformed with pEF-IRES-TMEPAI-WT-Flag and pcDNA3.1^+^ -HA-Hrs or pcDNA3.1^+^ -HA-STAM were extracted and immunoprecipitated with antibody to Flag-tag. The immunoprecipitates were analyzed by Western blot with antibodies to HA and Flag. (**C**) GST, GST-tagged cytoplasmic domain of TMEPAI (GST-TMEPAI) and ubiquitinated GST-TMEPAI (GST-TMEPAI (Ub)) were coupled to Glutathione Sepharose beads and incubated with extracts from HeLa cells transiently transfected with pcDNA3.1^+^ -HA-Hrs or pcDNA3.1^+^ -HA-STAM. Bound proteins were detected by Western blot with antibodies to HA (left). Protein ubiquitination was analyzed by Western blot with antibody against TMEPAI (right). (**D**) Immunofluorescence microscopy of A549 cell lines infected with Hrs-shRNA (Hrs-KD) lentivirus 4 days before being fixed and double labelled with antibodies to TMEPAI and either WGA-FITC, Golgin84, Lamp2 and EEA1 (early endosome marker). Scrambled shRNA was used as negative control. Scale bar, 10 μM. Hrs-shRNA lentivirus efficiency was accessed by Western blot with antibody against Hrs and quantitation (N = 3, **P < 0.01, ***P < 0.001). (**E**) Immunofluorescence microscopy of A549 cell lines infected with STAM-shRNA (STAM-KD) lentivirus 4 days before being fixed and double labelled with antibodies against TMEPAI (red) and either WGA-FITC, Golgin84, Lamp2 and EEA1 (green). Scrambled shRNA was used as negative control. Scale bar, 10 μM. STAM-shRNA lentivirus efficiency was accessed by Western blot with antibody against STAM and quantitation (N = 3, ***P < 0.001).

Ubiquitin binding proteins (UBPs) play an important role in the recognition and recruitment of ubiquitinated protein, and ubiquitinated proteins require binding to specific UBPs for targeting to relevant endomembrane compartment. To investigate whether UBPs serve as an adaptor to supervise TMEPAI trafficking, a series of UBPs including Hrs, STAM, GGA3, Tsg101 and Eap45 were selected to test the TMEPAI interaction by yeast two-hybrid analysis. The yeast two-hybrid results showed that TMEPAI interacted with Hrs and STAM (Fig. 5A). Interaction of TMEPAI between Hrs and STAM was further identified using an immunoprecipitation assay by co-expression of pEF-TMEPAI-WT-Flag and pcDNA3.1^+^ -HA-Hrs or pcDNA3.1^+^ -HA-STAM constructs respectively in HeLa cells (Fig. 5B). Furthermore, since ubiquitinated protein links with ubiquitin binding proteins through ubiquitin (Henne *et al*.^16^), GST-TMEPAI was first *in vitro* ubiquitinated to produce ubiquitinated GST-TMEPAI (Ub) for the GST pull-down assay, and the results indicated that ubiquitination is critical for TMEPAI interaction with Hrs and STAM (Fig. 5C). We next investigated the roles of Hrs and STAM in regulating TMEPAI intracellular transport. RNA interference of Hrs and STAM was performed in A549 cells and showed that TMEPAI was significantly distributed to the plasma membrane in either Hrs or STAM depleted cells, although with little colocalization with lysosomal marker Lamp2 and early endosome marker EEA1 (Fig. 5D and E). These results together demonstrate that ubiquitination is a sorting signal for TMEPAI lysosomal trafficking and that the ubiquitin binding proteins Hrs and STAM are involved in its lysosomal transport.

### Dynactin involvement in TMEPAI intracellular trafficking

**Figure 6:**
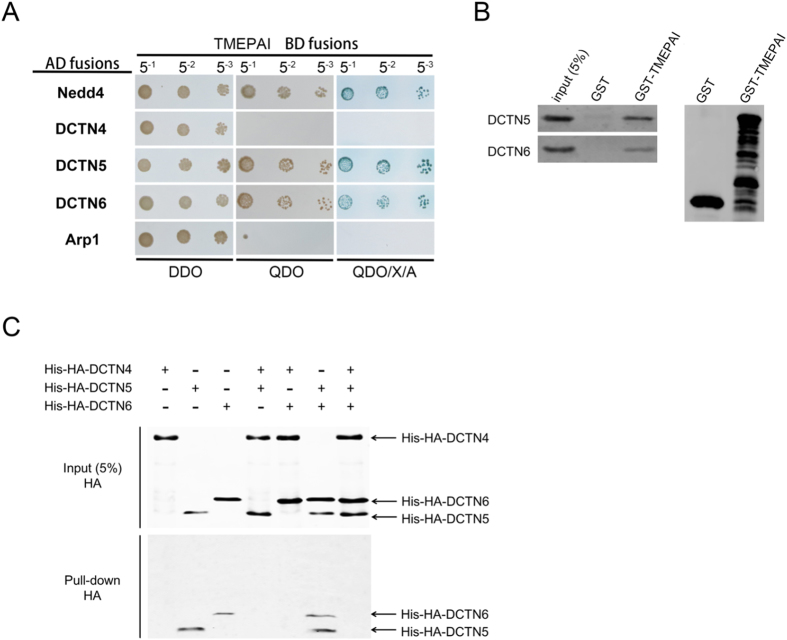
TMEPAI interacts with dynactin 5 and dynactin 6. (**A**) TMEPAI cytoplasmic domain (aa63-287) was tested for interaction with dynactin complex subunits dynactin 4 (DCTN4), dynactin 5 (DCTN5), dynactin 6 (DCTN6) and Arp1 using the yeast two-hybrid system. Yeast diploids were grown in liquid selective media. Diploids were titrated (5-1, 5-2, 5-3; total 3 μl) and patched on the DDO, QDO and QDO/X/A plates. The yeast co-expressing TMEPAI and Nedd4 was used as the positive control. (**B**) GST and the GST-tagged cytoplasmic domain of TMEPAI (GST-TMEPAI) were coupled to Glutathione Sepharose beads and incubated with His-HA-tagged recombinant proteins DCTN5 and DCTN6. Bound proteins were detected by Western blot with antibody to HA (left). GST, GST-TMEPAI proteins used for pull-down assay was analyzed by Western blot with antibody against GST (right). (**C**) Glutathione beads loaded with recombinant GST fused TMEPAI were incubated with His-HA-tagged recombinant DCTN4, DCTN5, DCTN6. The His-HA-DCTN4/5/6 in the reaction mixtures (input) and those bound to the GST-TMEPAI coupled beads (Pull down) were determined by Western blot using anti-HA antibody.

Dynactin acts as an important adaptor in combination with dynein and maintains dynein activity to drive membrane vesicles along microtubules for long-distance movement. The dynactin pointed-end complex consists of dynactin 4/p62, dynactin 5/p25 and dynactin 6/p27 and is proposed to be a cargo-targeting module. We initially found that TMEPAI interacted with dynactin 6 by yeast two-hybrid screening, thus we next set out to investigate whether TMEPAI interacts with the other two subunits (dynactin 4 and dynactin 5). Yeast two-hybrid experiments showed that TMEPAI interacted with both dynactin 5 and dynactin 6, but not with dynactin 4, or with another dynactin complex subunit Arp1, which forms the Arp1 rod for binding to membrane vesicles^37^, indicating that TMEPAI specifically combined with the point-end complex through dynactin 5 and dynactin 6 (Fig. 6A). Interaction of TMEPAI between dynactin 5 and dynactin 6 was further verified by GST pull-down assay using recombinant proteins (Fig. 6B). Since the integrity of dynactin point-end complex is essential for membrane binding, we addressed the question of whether the interaction between TMEPAI and dynactin 5, dynactin 6 might be reinforced when the point-end complex formed. We performed a GST pull-down assay to test the binding of GST-TMEPAI with different combinations of point-end complex subunits recombinant proteins. Interestingly, the interaction of TMEPAI with dynactin 5 and dynactin 6 was not strengthened with a combination of different subunits. In contrast, binding of TMEPAI to dynactin 5 and dynactin 6 was inhibited in the presence of dynactin 4 (Fig. 6C).

**Figure 7:**
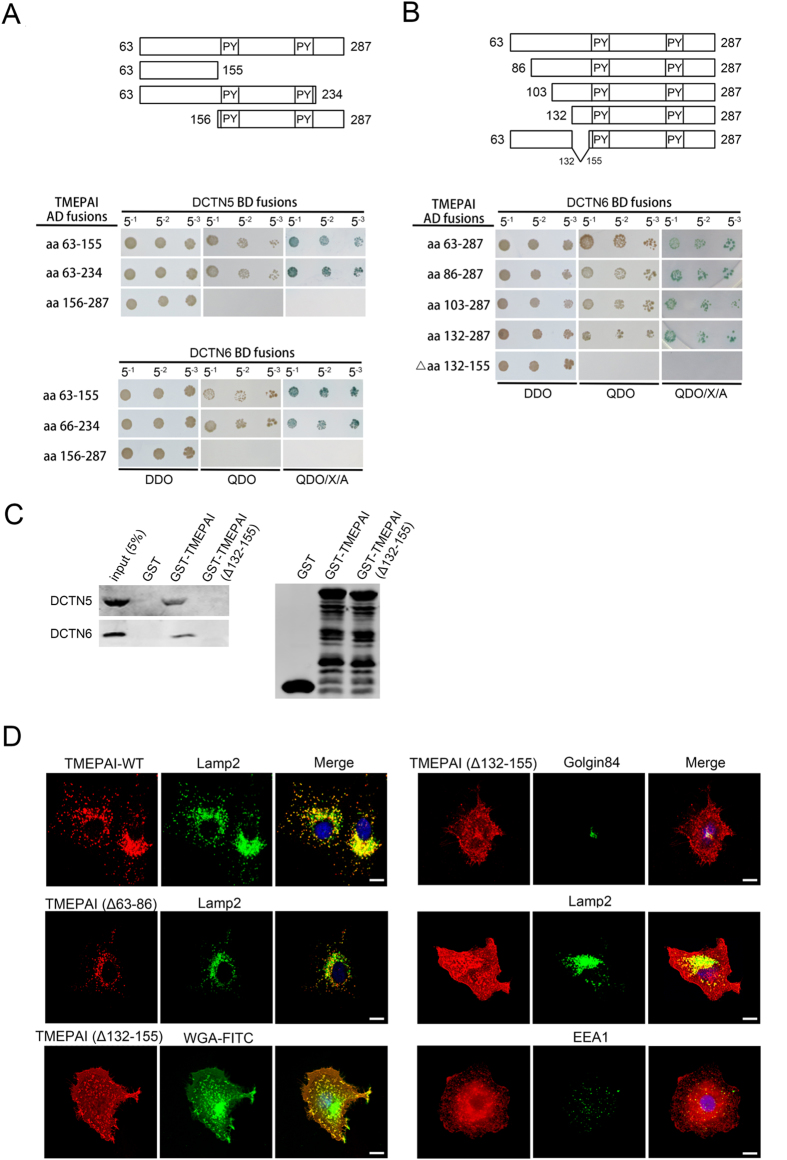
Interaction between TMEPAI and dynactin is required for TMEPAI lysosomal localization. (**A**,**B**) DCTN5 and DCTN6 fused to the GAL4 activation domain (AD fusions) were tested respectively for interaction in the yeast two-hybrid system with full-length, truncated and deleted versions of TMEPAI cytoplasmic domain fused to the GAL4 DNA-binding domain (BD fusion). Yeast diploids co-expressing GAL4 AD fusions and GAL4 BD fusions were grown in liquid selective media. Diploids were titrated (5^−1^, 5^−2^, 5^−3^; total 3 μl) and patched on DDO, QDO and QDO/X/A plates. (**C**) GST, GST-tagged cytoplasmic domains of TMEPAI (GST-TMEPAI) and GST-tagged cytoplasmic domains of TMEPAI with deletion of amino acids 132–155 (GST-TMEPAI (Δ132–155)) were coupled to beads and incubated with either His-HA-tagged recombinant proteins DCTN5 and DCNT6. Bound proteins were determined by Western blot with antibody to HA (left). GST, GST-TMEPAI and GST-TMEPAI (Δ132–155) proteins used for pull-down assay was analyzed by Western blot with antibody against GST (right). (**D**) Immunofluorescence microscopy of A549 cell lines transfected with Flag-tagged TMEPAI-WT, TMEPAI (Δ63–86) and (TMEPAI (Δ132–155) and labelled with antibodies to Flag (red), Golgin84, Lamp2, EEA1 and WGA-FITC (green). Scale bar, 10 μM.

To further identify the binding domain of TMEPAI with dynactin, we analyzed the homology sequence of TMEPAI protein and generated truncated versions of TMEPAI to test their interaction with dynactin 5 and dynactin 6 using the yeast two-hybrid system. The yeast two-hybrid experiments indicated that the N-terminal region of TMEPAI bound to both dynactin 5 and dynactin 6 (Fig. 7A), and further deletion mutation screening showed the aa132–155 domain was required for this interaction, as mutants lacking this region failed to bind dynactin 5 and dynactin 6 (Fig. 7B), but without affecting its binding to Nedd4 (data not shown). Furthermore, GST pull-down assay tests confirmed that GST-TMEPAI bound dynactin 5 and dynactin 6 but not the deletion mutant GST-TMEPAI (Δ132–155) (Fig. 7C). Moreover, we investigated whether the binding of TMEPAI to dynactin 5 and dynactin 6 contributed to its lysosomal localization. We generated TMEPAI mutants TMEPAI (Δ132–155) and TMEPAI (Δ63–86) with deletion of regions aa 132–155 and aa 63–86 respectively, and expressed these in A549 cells before double labelling with anti-Flag antibody and other membrane compartment markers. We observed that the mutant TMEPAI (Δ132–155) was significantly distributed to the plasma membrane, though with little colocalization with lysosomal marker Lamp2 and early endosome marker EEA1. The TMEPAI-WT and deletion mutant TMEPAI (Δ63–86) were localized to the lysosome normally (Fig. 7D). These results suggest that the aa132–155 domain of TMEPAI is required for its interaction with dynactin and lysosomal trafficking.

## Discussion

Each protein that enters the endo-lysosomal pathway passes a number of decision stations that determine the rest of its journey. Alternative pathways for the delivery of newly lysosomal proteins to the endo-lysosomal system demand multiple TGN exits with two lysosomal trafficking pathways options. Apart from the clathrin- and M6PR- mediated pathway, the biosynthetic pathways to the lysosome are poorly understood. Identification of distinct sorting mechanisms for direct targeting of lysosome membrane proteins (LMPs) or lysosomal hydrolases to the lysosome raises challenges in lysosomal trafficking research. Even a cross-link between lysosomal hydrolases and LMPs such as LIMP2 has been shown to interact with β-glucocerebrosidase (βGC) and mediate lysosome transport^38^. Blocking the interaction of TMEPAI with Nedd4 leads to translocation of TMEPAI to the plasma membrane, which raises the question of whether TMEPAI is delivered initially to plasma membrane before being internalized into the endo-lysosomal pathway. We found that endogenous TMEPAI remained in the lysosome location in cells treated with the endocytic inhibitor dynasore and genistein to inhibit the clathrin-dependent endocytosis. This suggests that TMEPAI is directly delivered to the endo-lysosome system from the Golgi, and it seems that TMEPAI is ubiquitinated before travelling into the endosome. A clathrin-dependent or -independent TGN exit pathway could be utilized by LMPs, Lamp1^39^ and LAPTM5^15^ separate from TGN via clathrin-coated vesicles (CCVs). Our study indicated that TMEPAI and its mutant targeted to their respective destination in a CCV-dependent pathway.

LMPs such as Lamp1 and Lamp2 are not modified with mannose 6 phosphate (M6P) groups and therefore are in the M6PR-independent sorting pathway. Instead, they undergo sorting as directed by their cytosolic tails that mediate both endo-lysosomal targeting and rapid endocytosis after travelling to the cell surface. Similar to other TGN sorting and endocytic signals, most lysosomal targeting signals belong to the YXXØ or [DE]XXXL[LI] types with certain features that make them functional for lysosomal trafficking^40^. In order to define whether TMEPAI trafficking to the lysosome is M6PR-mediated, we found that transport of TMEPAI was blocked in the Golgi under conditions of CI-M6PR depletion but not with CD-M6PR. However, the transport of TMEPAI (2YA) mutant to the plasma membrane was M6PR-independent. Although TMEPAI does not contain the typical residues for lysosome sorting signals described above, TMEPAI contains potential glycosylation sites according to the online N-glycosylation prediction software analysis (http://www.cbs.dtu.dk/services/NetNGlyc/), which suggests that glycosylation may serve as a sorting signal for TMEPAI lysosomal trafficking mediated by M6PR.

Ubiquitin, particularly monoubiquitin, has several proteasome independent functions in regulating membrane protein trafficking, including serving as a sorting signal on plasma membrane proteins for internalization and sorting into the endosomal vesicles for delivery into the lysosome. It also regulates biosynthetic membrane proteins trafficking from the Golgi to the endosomes or lysosomes^11^,^41^. The function of The Nedd4 family of E3 ubiquitin ligases in regulating endocytosis and the sorting of transmembrane proteins has been demonstrated in yeast and mammalian cells^13^,^14^. In mammalian cells, ubiquitination of GGA3 by Nedd4 regulates sorting of LAPTM5 from the Golgi to endosomes/lysosomes^15^. We found that interaction of TMEPAI with Nedd4 via its two PY motifs is responsible for its mono-ubiquitination. Biosynthetic and endocytic pathways converge at the early endosomes, which receives proteins coming from the TGN and the plasma membrane. In early endosomes, non-ubiquitinated proteins are recycled to the plasma membrane or directed to other membrane compartments, which may provide another explanation for the translocation to the plasma membrane of TMEPAI ubiquitination-defective mutants. In contrast, ubiquitinated proteins are sorted into intraluminal vesicles (ILVs) and develop into multivesicular bodies (MVBs)^11^,^42^. At the endosome, a series of proteins form ESCRT (endosomal sorting complex required for transport) to recruit ubiquitinated cargo to the endosome and sort into the interior of MVBs. These proteins are referred to as the ubiquitin binding proteins (UBPs) including Eps15, which operate at the plasma membrane to early endosome^43^. GGA3, mediates transport from the Golgi to the endosome^15^; Hrs/STAM, functions on interface between endosomes^44^,^45^; Tsg101, primary recruitment to late endosome^19^ and other ESCRT subunit complexes, all of which together are presumed to be involved in MVB formation. We found that TMEPAI directly interacts with both Hrs and STAM both by yeast two-hybrid and biochemical essays, and that either depletion of Hrs/STAM leads to redistribution of TMEPAI to the plasma membrane. A possible trafficking pathway of TMEPAI to the plasma membrane following UBP depletion involves TMEPAI being transported to the early endosome without recruitment by Hrs/STAM to ILVs, then recycling back to the Golgi and reaching the plasma membrane through the constitutive pathway.

Dynactin is responsible for dynein activity and drives cargo along microtubules for long distance movement. Our study showed that TMEPAI bound to both dynactin 5 and dynactin 6 simultaneously, but not dynactin 4 and the cargo-binding unit Arp1, indicating a specific association of TMEPAI with dynactin 5 and dynactin 6. Interestingly, dynactin 4 inhibited the binding of TMEPAI to dynactin 5 and dynactin 6, which suggested that dynactin 4 might function as a switch in modulating the binding and regulating the release of TMEPAI from the complex during its transport along the microtubule. Apart from dynein, dynactin also contributes to the activity of another microtubule-based motor protein, kinesin II^46^. Dynactin concentrates at the microtubule plus end and presents on various endo-membranes such as the early endosome and cell periphery^47^. Although dynactin is reported to conduct minus direction transport of microtubules, a recent study implied that dynactin could facilitate the mobility of adenovirus both towards and away from the nucleus^48^. In addition to binding dynein and maintaining its activity, dynactin functions as a cargo-targeting module by the point end complex, as dynactin 5 was discovered to be involved in endosome movement^24^ and dynactin 6 was found to assist polo-like kinase 1 target kinetochores during mitosis^49^. To investigate the role of dynactin in regulating TMEPAI intracellular trafficking, we identified the aa132–155 domain of TMEPAI as the dynactin binding region, and the dynactin-binding defective mutant TMEPAI (Δaa132–155) was found localized to plasma membrane. Although it has been reported that dynactin 5 and dynactin 6 are required for binding of dynactin and recruitment of dynein to the vesicles that recycle TfR to the cell surface, but not to late endosomes^25^, how this interaction with the dynactin complex regulates TMEPAI trafficking from the Golgi to lysosomes is still unclear and needs further investigation.

It has been reported that TMEPAI inhibits TGF-β signaling by blocking both R-Smad phosphorylation and by promoting degradation of TβR. This is not observed in the ubiquitination loss mutant TMEPAI (2YA)^30^,^31^, which suggests that ubiquitination contributes to TMEPAI lysosomal localization and the regulation of TGF-β signaling. A recent study has shown that TMEPAI promotes lysosome stability and autophagy and thus enhances cancer cells drug resistance to chemotherapeutics^32^. Protein expression, protein trafficking and subcellular localization, signaling transduction all co-ordinate to regulate normal cellular function. Our studies demonstrate that modification by ubiquitination and interaction with dynactin regulates TMEPAI intracellular trafficking to the lysosome. These findings extend the known roles of TMEPAI in tumorigenesis and might provide a novel strategy to modulate the transport and intracellular function of TMEPAI as a potential target for cancer treatment.

## Methods

### Materials and antibodies

All materials were from Sigma unless otherwise stated. Yeast two hybrid system kit and dropout solution screening medium were purchased from Clontech. Rabbit polyclonal antibody to TMEPAI was generated using the His-tagged cytoplasmic domain of TMEPAI as immunogen, antibody was affinity-purified against the GST-TMEPAI covalently coupled to Glutathione Sepharose (GE Healthcare). The specificity of the TMEPAI antibody was assessed by Western blot using the purified recombinant TMEPAI proteins and cell lysates^31^. Mouse anti-HA, Mouse anti-Flag and WGA-FITC were all from Sigma. Mouse anti-β-actin was from Tianjin Sungene Biotech. Sheep anti-Golgin84 and mouse anti-Lamp2 was from Abcam. Goat anti-EEA1 (N-19), mouse anti-TfR/CD71 (3B82A1), mouse anti-Rab7 (B-3), mouse anti-Clathrin HC (TD.1), rabbit anti-CI-M6PR (H-300), mouse anti-Hrs (D-3) and mouse anti-STAM (B-2) were from Santa Cruz Biotechnology. Fluorophore secondary antibodies were purchased from Invitrogen.

### Cell culture, transfection and stable cell lines

A549 cells were cultured in F-12K medium, supplemented with 10% fetal bovine serum (FBS), 100 U/ml penicillin and 100 μg/ml streptomycin. HeLa cells were maintained in DMEM medium containing 10% FBS, 100 U/ml penicillin, and 100 mg/ml streptomycin. Transfection was performed with Lipofectamine 2000 (Invitrogen) transfection reagent according to the manufacturer’s instructions. A549 cell lines stably expressing TMEPAI-2YA-Flag or TMEPAI-KR-Flag were obtained by transfection with pEF-IRES-puro-TMEPAI-2YA-Flag or pEF-IRES-puro-TMEPAI-KR-Flag recombinant plasmid and selected in 1 μg/ml puromycin for 2 weeks.

### Immunofluorescence microscopy

The cells were grown on coverslips and fixed in 4% PFA in PBS at room temperature (RT) for 20 min. Cells were quenched with 10 mM glycine, pH 8.5 (in PBS), and permeabilized with 0.1% TritonX-100 (in PBS) for 5 min at RT. Coverslips were incubated for 2 h at RT with primary antibodies diluted into PBS containing 3% BSA, washed three times with DPBS, incubated for a further 30 min at RT with fluorophore-conjugated secondary antibodies then DNA dye Hoechst 33342 (200 ng/ml) was mixed with the secondary antibodies. Coverslips were mounted in Mowiol and allowed to dry. Immunofluorescent images were viewed and analyzed using an Olympus FV1000 Confocal Microscope with the FV10-ASW 3.0 Viewer software.

### RNA interference

Lentiviral vectors expressing clathrin-shRNAs (clathrin-1: 5′-CCGGTAATCCAATTCGAAGACCAATCTCGAGATTGGTCTTCGAATTGGATTATTTTTG-3′); clathrin-2: 5′-CCGGCGGTTGCTCTTGTTACGGATACTCGAGTATCCGTAACAAGAGCAACCGTTTTTG-3′), CI-M6PR-shRNAs (CI-M6PR-1: 5′-CCGGCGGAGGAAATACTACCTCAATCTCGAGATTGAGGTAGTATTTCCTCCGTTTTTG-3′; CI-M6PR-2: 5′-CCGGCCGGACATCCAGCATCATATTCTCGAGAATATGATGCTGGATGTCCGGTTTTTG-3′), Hrs-shRNAs (Hrs-1: 5′-CCGGCCGCATGAAGAGTAACCACATCTCGAGATGTGGTTACTCTTCATGCGGTTTTTG-3′; Hrs-2: 5′-CCGGCTCACGTCCGGAGTAACACTACTCGAGTAGTGTTACTCCGGACGTGAGTTTTTTG-3′), STAM-shRNAs (STAM-1: 5′-CCGGTTAATACGTTGCTTACTTCACTAGCGCTAGTGAAGTAAGCAACGTATTAATTTTTTG-3′; STAM-2: 5′-CCGGATACATGGAATACATCGGATCTTCGCGAAGATCCGATGTATTCCATGTATTTTTTTG-3′) and the non-target shRNA control vector (SHC002) was obtained from Sigma and the knockdown level was tested by Sigma. The lentiviruses were produced according to the manufacturers’ manual. Cells were infected with lentiviruses for 4 days prior to the start of the experiments.

### *In vitro* ubiquitination assay

Purified His-HA-TMEPAI or His-HA-TMEPAI-2YA/KR proteins were incubated with recombinant E1 (Boston Biochem), recombinant UbcH5C (Boston Biochem), GST-Nedd4 and methylated ubiquitin or His-tagged ubiquitin (Boston Biochem) in reaction buffer (50 mM Tris-HCl, pH 7.4, 5 mM MgCl_2_, 0.3 mM DTT, 2 mM ATP) for 60 min at 30 °C. The reaction products were analyzed by SDS-PAGE and Western blot.

### *In vivo* ubiquitination assay

A549 cells stably expressing Flag-tagged TMEPAI-WT, TMEPAI-2YA or TMEPAI-KR were washed twice with PBS and lysed in the ice-cold lysis buffer (50 mM Tris-Cl, pH 8.0, 150 mM NaCl, 1% TritonX-100, 0.5% sodium deoxycholate, 0.1% SDS, 1 mM NaVO_4_, 10 mM NaF, and protease inhibitors). Lysates were left on ice for 40 min and centrifuged for 10 min at 12000 g at 4 °C. The supernatants were incubated with anti-Flag antibody for 2 h at 4 °C with rotation and further incubated for 2 h at 4 °C after addition of protein A/G Sepharose (Thermo Scientific). Immunoprecipitates were washed 3 times with lysis buffer and once with PBS, and then subjected to SDS-PAGE followed by Western blot analysis.

### Western blot

The cells were collected in RIPA buffer (50 mM Tris-Cl, pH 7.4, 150 mM NaCl, 1% TritonX-100, 0.5% sodium deoxycholate, 0.1% SDS, 1 mM NaVO_4_, 10 mM NaF, and protease inhibitors) for 40 min. Homogenates were clarified by centrifugation at 12000 g for 10 min at 4 °C and the supernatants were mixed with SDS-loading buffer and heated for 5 min at 95 °C. Samples were adjusted to equal protein concentration and volume, and subjected to SDS-PAGE. Separated proteins were transferred to PVDF membranes followed by blocking with 5% non- fat milk in PBS. The membranes were incubated with primary antibody, and subsequent secondary antibody. Proteins were visualized by Odyssey infrared laser imaging system (LI-COR Biosciences, Lincoln, NE, USA).

### Molecular biology and yeast two-hybrid analysis

Standard molecular biology techniques were used for all constructs. For the yeast two-hybrid assay, the full-length and truncated versions of *TMEPAI* cDNA lacking the trans-membrane domain were inserted into the NdeI and EcoRI sites of the pGBTKT vector. The full-length of human *Nedd4, Hrs, STAM, GGA3, Tsg101, Esp45, dynactin 4 (DCTN4*), *dynactin 5 (DCTN5*), *dynactin 6 (DCTN* 6) and *Arp1* cDNA were inserted into the pGADT7 vector (CLONTECH Laboratories, Inc.). The pGBKT7/TMEPAI and pGADT7 related plasmids were co-transformed into the yeast reporter strain Y187 on synthetic medium lacking leucine and tryptophan (double dropout, DDO) (low selection) and then patched onto synthetic medium lacking leucine, tryptophan, histidine, and adenine (quadruple dropout, QDO) (medium selection), as well as onto synthetic medium lacking leucine, tryptophan, histidine, and adenine with 40 μg/ml X-α-gal, 125 ng/ml Aureobasidin A (Aba A) (quadruple dropout with X-α-gal and Aba A, QDO/X/A) (high selection) according to the CLONTECH Laboratories, Inc. yeast protocol handbook.

### Protein expression and purification

Plasmids encoding His-HA-tagged TMEPAI-WT, TMEPAI-2YA, TMEPAI-KR, dynactin 4, dynactin 5, and dynactin 6 were transformed into BL21-CodonPlus (DE3)-RIL cells. Cells were induced with 0.5 mM IPTG overnight at 16 °C. Cells were lysed in ice cold lysis buffer (50 mM phosphate sodium buffer, pH 8.0, 150 mM NaCl, 5 mM β-mercaptoethanol, protease inhibitors) and the recombinant proteins were purified with Ni Sepharose beads (GE Healthcare).

### GST pull-down assay

Glutathione Sepharose beads coupled with 5 μg GST-fusion protein were incubated with the clarified cell lysates in RIPA buffer (50 mM Tris pH 7.4, 150 mM NaCl, 1% Triton X-100, 1% sodium deoxycholate, 0.1% SDS) with complete protease inhibitors at 4 °C for 3 h with rotation. The beads were washed four times with RIPA buffer and resuspended in SDS-PAGE sample buffer, bound and input proteins were subjected to SDS-PAGE and Western blot using indicated antibodies.

### Immunoprecipitation

A549 cell lines stably expressing Flag-tagged TMEPAI-WT, TMEPAI-2YA and TMEPAI-KR were extracted in IP buffer (20 mM Tris-Cl pH 7.4, 0.1 M KCl, 0.1 M NaF, 1 mM DTT, 1% TritonX-100) with complete protease inhibitors and clarified by centrifugation at 12000 g for 10 min, then incubated with anti-Flag M2 Magnetic Beads (Sigma M8823) for 4 h at 4 °C with gentle rotation. The beads were washed with IP buffer four times and eluted by boiling in SDS-PAGE sample buffer and analyzed by Western blot with appropriate antibodies.

### Statistical analysis

Statistical analysis was performed using the Student t-test with GraphPad Prism software, and P < 0.05 represented statistically significant difference.

## Additional Information

**How to cite this article:** Luo, S. *et al*. Ubiquitination and dynactin regulate TMEPAI lysosomal trafficking. *Sci. Rep.* **7**, 42668; doi: 10.1038/srep42668 (2017).

**Publisher's note:** Springer Nature remains neutral with regard to jurisdictional claims in published maps and institutional affiliations.
